# Th17.1 cell driven sarcoidosis-like inflammation after anti-BCMA CAR T cells in multiple myeloma

**DOI:** 10.1038/s41375-023-01824-0

**Published:** 2023-01-31

**Authors:** Alexander M. Leipold, Rudolf A. Werner, Johannes Düll, Pius Jung, Mara John, Emilia Stanojkovska, Xiang Zhou, Hannah Hornburger, Anna Ruckdeschel, Oliver Dietrich, Fabian Imdahl, Tobias Krammer, Stefan Knop, Andreas Rosenwald, Andreas Buck, Leif Erik Sander, Hermann Einsele, K. Martin Kortüm, Antoine-Emmanuel Saliba, Leo Rasche

**Affiliations:** 1 Helmholtz Institute for RNA-based Infection Research (HIRI), Helmholtz-Center for Infection Research (HZI), Würzburg, Germany; 2 Mildred Scheel Early Career Center, University Hospital of Würzburg, Würzburg, Germany; 3 Department of Nuclear Medicine, University Hospital Würzburg, Würzburg, Germany; 4 Department of Internal Medicine 2, University Hospital of Würzburg, Würzburg, Germany; 5 Department of Internal Medicine 1, University Hospital of Würzburg, Würzburg, Germany; 6 Institute of Pathology, University of Würzburg, Würzburg, Germany; 7 Charité - Universitätsmedizin Berlin, Corporate Member of Freie Universität Berlin and Humboldt-Universität zu Berlin, Department of Infectious Diseases, Respiratory Medicine and Critical Care, Berlin, Germany; 8 Berlin Institute of Health, Center for Regenerative Therapies, Berlin, Germany

**Keywords:** Cancer immunotherapy, Immunotherapy, Autoimmune diseases

## Abstract

Pseudo-progression and flare-up phenomena constitute a novel diagnostic challenge in the follow-up of patients treated with immune-oncology drugs. We present a case study on pulmonary flare-up after Idecabtagen Vicleucel (Ide-cel), a BCMA targeting CAR T-cell therapy, and used single-cell RNA-seq (scRNA-seq) to identify a Th17.1 driven autoimmune mechanism as the biological underpinning of this phenomenon. By integrating datasets of various lung pathological conditions, we revealed transcriptomic similarities between post CAR T pulmonary lesions and sarcoidosis. Furthermore, we explored a noninvasive PET based diagnostic approach and showed that tracers binding to CXCR4 complement FDG PET imaging in this setting, allowing discrimination between immune-mediated changes and true relapse after CAR T-cell treatment. In conclusion, our study highlights a Th17.1 driven autoimmune phenomenon after CAR T, which may be misinterpreted as disease relapse, and that imaging with multiple PET tracers and scRNA-seq could help in this diagnostic dilemma.

## Introduction

CAR T-cell therapy is establishing itself as a new standard of care in relapsed and refractory multiple myeloma (RRMM), and the field is moving towards use of this modality in early treatment lines [1]. For example, anti-BCMA Ide-cel provides a median progression-free survival of 12.1 months in a heavily pretreated and triple-refractory patient population [2]. Yet, there are a number of open issues including the lack of data on optimal follow-up assessments and autoimmune phenomena post CAR T therapy. In this context it is worth mentioning that patients with autoimmune diseases were usually excluded from trials and the impact of these novel treatments on pre-existing conditions is unknown.

Appreciating the high incidence of extramedullary lesions and patchy disease patterns seen in the RRMM setting, whole body ^18^F-Fluorodeoxyglucose (FDG) PET/CT imaging is increasingly used in addition to serological M-protein testing and bone marrow aspirates for staging prior to and after CAR T infusion. Whether FDG is the best tracer in this setting has yet to be determined, as the technique is being based not only on the quantification of increased glucose uptake by tumor cells but also by others such as inflammatory cells [3]. Given the immunologic mode of action of CAR T-cell treatment, atypical response patterns related to CAR T-cell expansion and local inflammation could challenge image interpretation and subsequent response assessment. While data on MM have still to emerge, two pilot studies in lymphoma described pseudo-progression in patients after CD19 directed CAR T-cell therapy [4, 5]. Pseudo-progression refers to an increase in the target lesion size due to influx of immune cells, and is a well-known event after checkpoint blockade therapy, seen in up to 10% of patients [6, 7]. Other checkpoint inhibitor-related adverse events include sarcoidosis-like reactions, presenting as pulmonary nodules and mediastinal lymphadenopathy [8]. In general, the incidence of immune-related changes after CAR T therapy in MM is unknown, but strategies to discriminate progression from immune-related phenomena are required as these products become more commonly used.

Here, we report on a sarcoidosis-like flare-up after anti-BCMA CAR T-cell product Ide-cel and present our strategy to discriminate between autoimmune phenomena and true relapse using multiple PET tracers along with single-cell RNA-sequencing (scRNA-seq).

## Results

### Case presentation

**Fig. 1:**
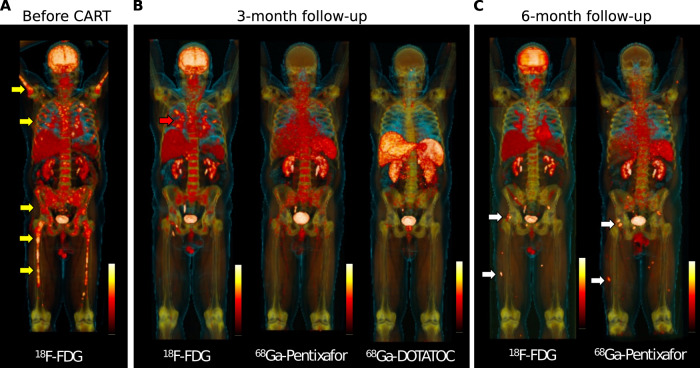
Whole body PET imaging at baseline and during follow-up using multiple PET tracers. **A** FDG-PET at baseline shows multiple focal lesions at the axial and appendicular skeleton (yellow arrows). **B** Follow-up assessment at 3-month after CAR T infusion shows full resolution of focal lesions, but residual FDG uptake located to the lung (red arrow). Matched-simultaneous CXCR4-targeted Pentixafor and DOTATOC PET did not show focal uptake. **C** Matched-simultaneous FDG and CXCR4 PET 6 months after CAR T depicted new focal lesions at the pelvis and lower limbs in line with relapse (white arrows).

A 61-year-old Caucasian male was diagnosed with IgA kappa MM with osteolytic bone disease, anemia, and 80% bone marrow infiltration by malignant plasma cells. In addition, biopsy-proven extramedullary disease (EMD) was found located to both lungs, explaining months of violent coughing prior to presentation. Fluorescent in situ hybridization revealed high-risk cytogenetics including t(14;20), amp(1q), and del(1p). The patient responded to induction therapy using bortezomib, cyclophosphamide, dexamethasone, but stem cell harvest was skipped due to the COVID-19 pandemic. A bortezomib based bridging therapy failed, M-protein levels increased and salvage therapy was administered using daratumumab, pomalidomide, dexamethasone to which the patient was refractory. Given the limited options at this stage, the patient was treated with the anti-BCMA CAR T-cell product ide-cel. PET imaging using multiple tracers was conducted at baseline, 3-month follow-up, and 6-month follow-up (Fig. 1A–C, Supplementary Fig. 1A–F). Baseline FDG PET scan showed multiple focal bone lesions at the axial and appendicular skeleton as well as pulmonary manifestations with mediastinal lymphadenopathy (Fig. 1A, Supplementary Fig. 1A). The patient responded to carfilzomib-containing bridging therapy [9], and the target dose of 450 × 10^6^ CAR T cells was infused. The patient experienced rapid-onset cytokine release syndrome (CRS) grade 1, which was successfully treated with repetitive doses of tocilizumab. Respiratory symptoms fully resolved during bridging therapy but came back 10 days after CAR T infusion. Disease assessment was performed at 3 months of follow-up revealing undetectable MRD in a bone marrow aspirate and full resolution of focal bone lesions but still disseminated FDG uptake at the lung and mediastinal lymph nodes. To narrow the differential diagnoses down, we performed a PET using ^68^Ga-DOTA(0)-Phe(1)-Tyr(3)-octreotide (^68^Ga-DOTATOC) that usually detects sarcoidosis with high sensitivity and specificity [10], which was negative. Negative results were also seen for ^68^Ga-Pentixafor (binds to CXCR4), which is an alternative tracer to FDG in the detection of malignant plasma cells [11] (Fig. 1B, Supplementary Fig. 1B–D). Bronchoalveolar lavage (BAL) along with endobronchial ultrasound guided biopsy was done, showing a CD4 to CD8 ratio of 2.1 on cytology and noncaseating granuloma formation in the biopsy without evidence of residual MM cells. Infectious disease workup included microscopy, culture, ELISA and PCR for fungal and mycobacterial pathogens, which was negative.

### Single-cell RNA-sequencing reveals presence of Th17.1 cells in the bronchoalveolar lavage

**Fig. 2:**
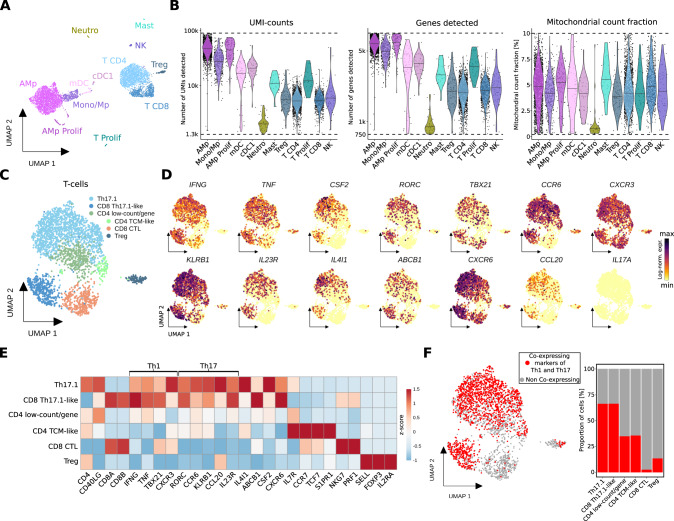
Single-cell analysis of bronchoalveolar lavage of a MM patient with FDG-avid pulmonary manifestation on PET-imaging suggests Th17.1-driven autoimmune phenomenon following anti-BCMA CAR T treatment. **A** UMAP embedding of 6,042 single-cell transcriptomes from two technical replicates from BAL (bronchoalveolar lavage) of a MM patient at three months follow-up after CAR T therapy (CART-BAL). Cell type annotation was based on expression of canonical marker genes (Supplementary Fig. 2D). **B** Data quality metrics across cell types in CART-BAL depicted in violin plots. Dashed lines represent thresholds that were used for quality control filtering. For UMI-counts and genes detected, log10-scale is shown. Lines in violins show medians per cell type. **C** UMAP embedding of 2,778 T-cells (T CD4, T CD8, Treg from **A**) from CART-BAL colored by subset annotation. **D** Log-normalized gene expression of Th1-associated genes (*IFNG*, *TNF*, *TBX21*, *CXCR3*), Th17-associated genes (*RORC*, *CCR6*, *KLRB1*, *IL23R*, *CCL20*) and further selected genes (*CSF2*, *IL4I1*, *ABCB1*, *CXCR6*) that together characterize Th17.1-cells and *IL17A* color-coded and projected onto the UMAP embedding from **C**. **E** Heatmap showing the z-score of mean log-normalized expression of selected genes per T-cell subset identified in **C**. **F** Co-expression of Th1-associated (*IFNG*, *TNF*, *TBX21*, *CXCR3*) and Th17-associated (*CCL20*, *IL23R*, *RORC*, *CCR6*) genes in individual cells was assessed and projected onto the UMAP embedding as well as depicted as bar plot. Cells were deemed co-expressing when at least one Th1- and one Th17-associated gene was detected. AMp alveolar macrophage, Mono monocyte, Mp macrophage, CTL cytotoxic T-lymphocyte, TCM central memory T-cell.

To unravel the biological underpinnings, we performed scRNA-seq on the BAL sample including 6,042 cells (henceforth called CART-BAL), of which none was identified as plasma cell (Fig. 2A, Supplementary Fig. 2A–D, Supplementary Table 1). Quality metrics were satisfactory across identified cell types (Fig. 2B). As the CD4:CD8 T-cell ratio in both, cytology and scRNA-seq was increased (Supplementary Fig. 2E), we decided to perform a focused transcriptomic analysis of T-cells. They were mainly CD4-positive cells exhibiting simultaneous expression of genes associated with Th1 T-cells (*TBX21*, *CXCR3*, *IFNG*, *TNF*) and genes associated with Th17 T-cells (*RORC*, *IL23R*, *CCR6*, CCL20, *KLRB1*), indicative for a Th1-polarized Th17 phenotype (Fig. 2C–E, Supplementary Fig. 3A–D, Supplementary Table 2). Furthermore, these cells expressed *IL4I1*, *ABCB1*, and *CSF2* [12–14], while *IL17A*, *IL17B*, *IL17E* (*IL25*), and *IL17F* were not expressed (Fig. 2D, E, Supplementary Fig. 3C). Of note, the terminology for these Th1-polarized Th17 cells in the literature is not uniform. T-cell phenotypes with similar characteristics have been coined Th17.1, Th1/17, nonclassical Th1, and extinguish (ex)Th17 [15–17]. They have been described to be pro-inflammatory and pathogenic and are implicated in several autoimmune diseases, including sarcoidosis [13, 14, 17–19]. To stay consistent with previous reports in pulmonary sarcoidosis, in which the majority of cells produced IFNγ but not IL17, we will refer to them as Th17.1 cells [17]. We also identified CD8-positive cells exhibiting gene expression resembling Th17.1 cells **(**Fig. 2C–E**)**. Consequently, we termed this cluster CD8 Th17.1-like. Also CD8-positive cells with Th17-like features and plasticity towards *IFNG* expression have been linked to autoimmune diseases [20–23]. Th17.1 and CD8 Th17.1-like cells expressed a number of pro-inflammatory cytokines, including *IFNG*, *TNF*, *CSF2* (*GM-CSF*), *CCL20*, and *IL26*, highlighting their polyfunctional nature [15, 24] (Fig. 2D, E, Supplementary Fig. 3C). To validate the identified Th17.1 cells as genuine, we examined co-expression of four Th1-associated (*IFNG*, *TNF*, *TBX21*, *CXCR3*) and four Th17-associated (*CCL20*, *IL23R*, *RORC*, *CCR6*) genes in individual cells. They were considered co-expressing when at least one Th1- and one Th17-associated gene was detected in a single cell, and we confirmed co-expression in the majority of Th17.1 and CD8 Th17.1-like cells. (Fig. 2F). As we used 3’ chemistry for scRNA-seq, we were not able to determine if anti-BCMA CAR T-cells were present in CART-BAL.

### Transcriptional similarities between post CAR T pulmonary lesions and sarcoidosis

**Fig. 3:**
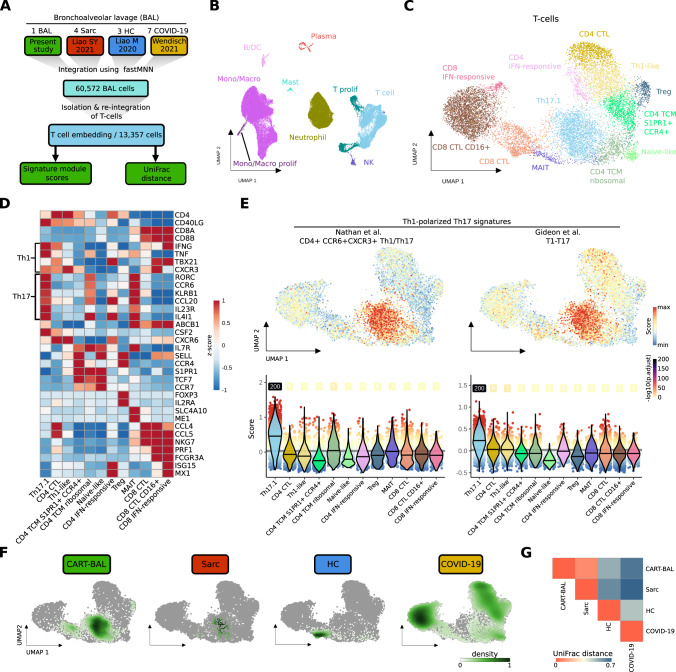
scRNA-seq data integration reveals phenotypic similarities between T-cells from CART-BAL and patients suffering from pulmonary sarcoidosis. **A** Schematic depicting the integration workflow of BAL data from present study with BAL data from sarcoidosis, healthy controls and COVID-19 ARDS using fastMNN. T-cells were isolated and re-integrated. Signature module scores and UniFrac distances were computed. **B** UMAP embedding of 60,572 cells from the integrated datasets colored by cell type. **C** UMAP embedding of 13,357 T-cells isolated from the integrated datasets and re-integrated, colored by T-subsets. **D** Heatmap showing the z-score of mean log-normalized expression of selected genes per T-subset. **E** Cell-based gene set module scores of two Th1-polarized Th17 gene signatures. Top: Projected onto the UMAP embedding from **C**. Bottom: depiction as violin plots across T-subsets (violin color). Lines in violins show median scores per T-subset. Grey lines indicate the average scores across all T-cells. Dot color specifies the signature module score and numbers specify -log10 transformed adjusted *p*-values (one-sided Wilcoxon rank-sum test against the average; −log10(p.adjust) with value ‘infinite’ (*p*.adjust = 0) were set to 200). **F** Kernel density estimations of cells from the four conditions (CART-BAL, Sarc, HC, COVID-19) used in data integration shown as UMAP overlay for individual conditions. **G** Heatmap showing the correlations between T-cells of integrated conditions by UniFrac distances. BAL bronchoalveolar lavage, Sarc sarcoidosis, HC healthy control, Mono/Macro monocytes, and macrophages, CTL cytotoxic T-lymphocyte, TCM central memory T-cell, MAIT mucosal-associated invariant T-cell.

To further explore the role of Th17.1 cells in CART-BAL, we integrated the data with additional publicly available scRNA-seq datasets (Fig. 3A, Supplementary Fig. 4A, B, Supplementary Table 1). First, we used data from three healthy control BALs (HC in Fig. 3), in order to confirm our findings to be truly related to pathological conditions [25]. Next, we used BAL data from seven patients suffering from severe COVID-19 acute respiratory distress syndrome (ARDS) with profound profibrotic lung remodeling (COVID-19 in Fig. 3), constituting a control for overwhelming immune responses to an infectious trigger [26]. Last, we used BAL data from four pulmonary sarcoidosis patients (Sarc in Fig. 3), as localisation of lesions and their appearance on FDG-PET was reminiscent of sarcoidosis [27]. We isolated and re-analysed T-cells and identified several subsets based on their gene expression profile, including Th17.1 cells, (Fig. 3B–D, Supplementary Fig. 5A–C, Supplementary Fig. 6A, B, Supplementary Table 2). Th17.1 cells were validated using module scores built on two published gene signatures of Th1-polarized Th17 cells [28, 29] (Fig. 3E, Supplementary Fig. 6C, Supplementary Table 1). T-cells from sarcoidosis patients predominantly localized to the same UMAP space as CART-BAL T-cells, in line with profound phenotypic similarities (Fig. 3F). To further explore the similarity between post CAR T pulmonary changes and sarcoidosis, we applied UniFrac distance calculation [30]. Here, a distance of 0 refers to a situation in which two conditions have exactly the same composition while a distance of 1 refers to completely different compositions. A UniFrac distance of 0.09 confirmed profound overlap between the two pathological conditions (Fig. 3G). Of note, the module scores based on the two Th1-polarized Th17 gene signatures were high in sarcoidosis Th17.1 cells (Supplementary Fig. 6D). To further interrogate similarities between CART-BAL and sarcoidosis T-cells, we projected skin derived single T-cell transcriptomes from three cutaneous sarcoidosis patients onto the integrated BAL T-cell embedding [31] (Supplementary Fig. 7A–D). Here, a substantial proportion of cells mapped onto the UMAP space of Th17.1 cells (Supplementary Fig. 7A). As the mapping requires confirmation, we performed three more validation steps. First, label transfer predicted the cells as Th17.1 with high confidence (Supplementary Fig. 7B); second, Th1- and Th17-associated genes were expressed (Supplementary Fig. 7C); and third, Th1-polarized Th17 signature module scores were highest within these cells (Supplementary Fig. 7D), further validating our observation. We did not analyse the peripheral blood and thus were not able to detect peripheral hyper-activation of T-cells as it was observed in sarcoidosis, previously [32].

While we observed aberrant T-cells in the CART-BAL dataset, the macrophage compartment in CART-BAL, sarcoidosis, and HC was mainly comprised of unremarkable tissue resident alveolar macrophages. A pro-fibrotic macrophage phenotype (*CD163*/*LGMN*-Mp), expressing high levels of *SPP1*, recently identified in severe COVID-19 ARDS and IPF (idiopathic pulmonary fibrosis), was absent from CART-BAL, sarcoidosis and HC BALs [26, 30, 33] (Supplementary Fig. 8A–C). The same holds true for an accumulation of neutrophils that was only present in patients with severe COVID-19 ARDS, consistent with acute infection in those patients (Supplementary Fig. 5C).

Together, our data suggest a non-infectious, Th17.1 T-cell driven sarcoidosis-like autoimmune phenomenon located in the lungs following anti-BCMA CAR T-cell therapy. Of note, not a single plasma cell transcriptome was observed in CART-BAL, excluding resistant disease to cause the FDG signal in PET imaging.

### Discriminating myeloma relapse from sarcoidosis using PET imaging

**Fig. 4:**
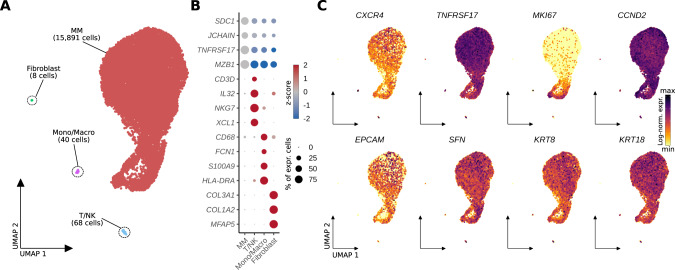
scRNA-seq analysis of a soft-tissue lesion in the lower limb six months after anti-BCMA CAR T-cell therapy showed expression of epithelial-associated genes in multiple myeloma cells. **A** UMAP embedding of 16,007 single cells from an extramedullary soft-tissue lesion colored by cell type. **B** Dotplot showing scaled expression (color) of respective marker genes per cell type. Dot size depicts the percentage of non-zero expressing cells. **C** Log-normalized gene expression of *CXCR4*, the CAR T target antigen *TNFRSF17* (*BCMA*), proliferation marker *MKI67*, *CCND2* (marking malignancy), as well as epithelial cell associated genes (*EPCAM*, *SFN*, *KRT8*, *KRT18*) color-coded and projected onto the UMAP embedding from **A**. Mono/Macro monocytes/macrophages.

At 6 month follow-up, the patient was still coughing. CAR T-cell numbers in the peripheral blood declined from initially 10% to 0.05% of PBMCs, and humoral immunity started to recover. FDG PET showed the known sarcoidosis-like pulmonary changes, but also revealed a number of new soft-tissue lesions located to the musculature of both lower limbs, suspicious for extramedullary relapse. In contrast to the previous scans, these lesions were CXCR4 positive (Fig. 1C, Supplementary Fig. 1E, F). We biopsied one of the lesions and performed scRNA-seq, revealing 99.3% of malignant plasma cells with strong *TNFRSF17* (*BCMA*) and *CCND2* expression of which a considerable amount was in a proliferative state (Fig. 4A–C). *CXCR4* expression was detected on RNA level and expression was confirmed on protein level using flow cytometry, explaining PET positivity for CXCR4 (Supplementary Fig. 9B, Supplementary Fig. 9A). Intriguingly, a number of unexpected genes were expressed, usually seen in epithelial cells such as *EPCAM*, *SFN*, *KRT8* and *KRT18*, suggesting a plasma-epithelial transition (Fig. 4C). EPCAM expression was also confirmed on protein level using flow cytometry (Supplementary Fig. 9A). We compared transcriptomes from the extramedullary lesion (EMD) to two publicly available single-cell datasets including one post-CAR T relapse [34] and 13 newly diagnosed MM cases [35]. This plasma-epithelial gene expression was unique to our case (Supplementary Fig. 9B). Whether expression of these genes was linked to extramedullary growth or even to progression from CAR T-cell therapy, has yet to be determined. The patient was later salvaged with a CXCR4-directed endo-radiotherapy (ERT), using a single intravenous injection of ^90^Y- labelled Pentixather (7305 GBq), followed by high-dose melphalan and autotransplant two weeks after ERT [36]. The patient received complete remission, but relapsed 4 months after ERT and was refractory to salvage chemotherapy. Of note, respiratory symptoms completely resolved following high-dose inhalative steroids 10 weeks after autotransplant.

## Discussion

We hypothesize that CRS after CAR T-cell infusion triggered expansion of pathogenic Th17.1 T-cells in the lung leading to sarcoidosis-like manifestations. Th1-polarized Th17 cells are key effectors of autoimmune inflammation [12] including inflammatory bowel disease [13], multiple sclerosis [37], and sarcoidosis [17]. T-cells with Th17.1 characteristics have been described to be enriched in the bone marrow of MM patients [38]. Of note, our patient suffered from rare pulmonary EMD at the time of diagnosis. Thus, preexistence of Th17.1 T-cells in the lung prior to CAR T-cell treatment is likely and could represent a risk factor for sarcoidosis-like reactions. Unmasking of autoimmune diseases is a known side effect after checkpoint inhibitors [39, 40], and here we describe a similar phenomenon after CAR T therapy.

PET imaging using multiple tracers is a noninvasive approach to decode tumor heterogeneity in MM [41]. Using the three tracers FDG, DOTATOC, and CXCR4-targeted Pentixafor, we were able to discriminate between autoimmune inflammation and MM relapse. In light of the plethora of novel tracers currently entering nuclear medicine [41, 42], PET imaging beyond FDG is a promising tool in the follow-up assessment of CAR T treated patients allowing for discrimination between inflammation and true relapse. Of note, in the absence of known pulmonary MM manifestations, sarcoidosis on FDG PET/CT is a known pitfall, with typical imaging features on PET, and usually does not need confirmation using alternative radiotracers. However, our case study is an illustrative example on how multi tracer PET imaging could help to narrow down the differential diagnosis. Another potential biomarker helping to distinguish MM progression from immune-mediated changes is soluble (s)BCMA. A limitation, however, could be that sBCMA clearance takes several months and flare up phenomena typically occur early after initiation of immunotherapy. Moreover, we and others have reported on MM progression after anti-BCMA CAR T-cell therapy lacking sBCMA increase due to clonal biallelic *BCMA*-loss [34, 43].

Finally, we report the case of a BCMA positive relapse after anti-BCMA CAR T. While antigen loss is a rare tumor-intrinsic mechanism of resistance [34, 43], the biology underlying relapse with preserved antigen expression is poorly understood. In our case, relapse occurred along with recovery of non-involved immunoglobulins, suggesting loss of CAR T-cell function and subsequent outgrowth of residual MM cells. Larger trials are warranted to investigate whether the plasma-epithelial signature that we described contributed to relapse. Appreciating the lack of activity of CAR T cells in carcinoma and the limited duration of response to CAR T in EMD compared to patients without EMD [44], plasma-epithelial transformation could alter CAR T-cell activity and confer a mechanism of resistance.

In conclusion, we demonstrated a clinically relevant autoimmune phenomenon after CAR T-cell therapy that can be delineated using scRNA-seq and PET imaging.

## Methods

### Radiotracer synthesis, image protocols and interpretation

We used a fully automated synthesis module (Scintomics, Fürstenfeldbruck, Germany) to synthesize ^68^Ga-Pentixafor, as described previously [36]. ^18^F-FDG and ^68^Ga-DOTATOC were prepared as described previously [45, 46]. PET/CTs were carried out using a dedicated PET/CT scanner (Siemens Biograph mCT 64 or mCT 128; Siemens Medical Solutions, Germany). Prior to administration of ^18^F-FDG, patients fasted for at least 6 h with blood glucose levels below 160 mg/dl at time of scan. For ^68^Ga-Pentixafor and ^68^Ga-DOTATOC PET/CT, fasting was not required. Spiral CT with or without intravenous contrast was conducted and included a field of view from the base of the skull to the proximal thighs. PET emission data were acquired in three-dimensional mode (200 × 200 matrix, 2–3 min emission time per bed position). After decay and scatter correction, PET data were then reconstructed iteratively using the algorithm provided by Siemens Esoft (Siemens, Erlangen, Germany). Image interpretation was conducted on a visual basis and carried out by an expert reader (RAW).

### Sample preparation and scRNA-seq processing

CART-BAL sample was obtained at 3-month follow-up, diluted in PBS and filtered through a 40 μm mesh resulting in a single cell suspension. EMD sample was obtained at 6-month follow-up by ultrasound guided push biopsy. The biopsy was filtered with a 100 μm strainer followed by tissue digestion using Collagenase A (10 mg/mL), Collagenase D (10 mg/mL), DNAse I (2 mg/mL). Digestion was stopped with EDTA (100 mM) followed by a second filtering through a 70 μm mesh resulting in a single cell suspension.

Obtained single cell suspensions were adjusted to a concentration of 400 cells/μl and 1000 cells/μl for CART-BAL and EMD, respectively, and loaded into the 10x Chromium controller for scRNA-seq. Following the detailed protocol provided by 10x genomics, single cell 3’ reagent kit v3.1 was used for reverse transcription, cDNA amplification and library construction. Libraries were quantified using QubitTM 2.0 Fluorometer (ThermoFischer) and quality was checked by 2100 Bioanalyzer with High Sensitivity DNA kit (Agilent). S2 flow cells were used for sequencing on a NovaSeq 6000 sequencer (Illumina). CART-BAL has been run in duplicate to obtain technical replicates.

### Single-cell RNA-seq analysis

#### scRNA-seq analysis of CART-BAL dataset

Raw sequencing data was demultiplexed and quality-checked using the CellRanger (v.3.1.0) ‘mkfastq’ script. Alignment and transcript quantification was performed using the CellRanger ‘count’ script against the GRCh38 (Ensembl93) human genome assembly. Ambient RNA signals were removed using SoupX to mitigate potential effects of RNA released from dead or ruptured cells [47]. The obtained SoupX-adjusted count matrices were loaded into R (4.1.0) and downstream analysis was performed using the Seurat R package (v4.1.1) [48]. Low quality transcriptomes, as well as epithelial cells (based on expression of *EPCAM*) were removed. Quality filtering thresholds for the number of detected genes, number of detected unique RNA molecules (UMIs) and fraction of mitochondrial UMI-counts are available in Supplementary Table 1. Count data was log-normalized (Seurat function NormalizeData), highly variable features were identified (Seurat function FindVariabeFeatures, n.features = 5000) and scaled (Seurat function ScaleData). Principal component analysis (PCA) was calculated using RunPCA function of Seurat based on highly variable genes. A two-dimensional embedding was computed with the uniform manifold approximation and projection (UMAP) algorithm [49] using the first 45 PCA-dimensions (Seurat function RunUMAP). Nearest neighbor graph construction was performed using the Seurat function FindNeighbors (dims = 1:45). Unsupervised clustering was applied using the Louvain algorithm implemented in Seurat (Seurat function FindClusters) with the resolution parameter set to 1.8.

For analysis of CART-BAL T-cells respective clusters (T CD4, TCD8, Treg) were isolated from the dataset using the subset function and the subset was reanalysed repeating basic Seurat steps (described above) based on 1000 highly variable features, the first 15 PCA-dimensions, and clustering resolution parameter set to 0.7.

#### Analysis of integrated BAL datasets

BAL Single-cell transcriptomes generated in this study (CART-BAL) and three previous studies, including BAL samples from four pulmonary sarcoidosis patients [27], three healthy donors [25] and seven patients with severe COVID-19 acute respiratory distress syndrome (ARDS) with profound fibrotic remodeling [26], were integrated into a single embedding. The integration workflow is sketched in Supplementary Fig. 5. Raw sequencing data were acquired from Wendisch et al. [26] (COVID-19) and Liao M. et al. [25] (HC). Alignment was performed using a common CellRanger version (v3.1.0) and a common reference (GRCh38, Ensembl93) to avoid confounding factors from this step, as the recently pre-printed human lung cell atlas has demonstrated the CellRanger version to constitute a key factor of variance, especially in the T-cell lineage [50]. After obtaining count-matrices, SoupX was used to remove ambient RNA signals in each dataset [47]. As Sarcoidosis BAL scRNA-seq data was not available as raw sequencing data due to patient privacy restrictions, count level data were acquired from this study (Sarc). These data were aligned with CellRanger v3.1.0 to reference GRCh38 Ensembl93 in the original study [27]. The datasets were further processed, and quality control was performed using Seurat v4.1.1 based on customized thresholds for the number of detected genes, number of detected unique RNA molecules (UMIs) and fraction of mitochondrial UMI-counts (Supplementary Table 1). In addition, epithelial and erythrocyte clusters were removed. All datasets were merged, and initial basic Seurat analysis was performed using 3000 highly variable genes and 55 first PCA-dimensions. As confounding covariates “patient” for the CART-BAL datasets (two replicates/datasets, one patient), COVID-19 datasets (7 datasets, 7 patients), and sarcoidosis datasets (4 datasets, 4 patients) as well as “donor” for healthy controls (3 datasets, 3 donors) were identified (Supplementary Fig. 5B). Data integration correcting for these covariates was performed using the fastMNN implementation of the Bioconductor batchelor package based on the 3000 highly variable genes [51]. The fastMNN approach for data integration was chosen as it was one of the top-performers in a recent benchmark by Luecken et al., especially in the human immune cell task [52]. For computation of the UMAP embedding and nearest neighbor graph construction 55 first MNN-dimensions were used. Unsupervised clustering, based on the nearest neighbor graph, was performed using the Louvain algorithm with the resolution parameter set to 0.4.

Of note, dataset HC4 from the Liao M. et al. paper was excluded due to patient privacy restrictions. In the original study alignment was performed using a different CellRanger version and reference as desired for the integration pipeline in the present study [33].

T-cells and monocyte/macrophage (Mono/Macro) cells were separated from the integrated dataset and reanalysed, repeating integration as described above. For T-cells and monocyte/macrophage subsets 3000 and 1000 highly variable genes, 55 and 25 first PCA-dimensions, 55 and 25 first MNN-dimensions, and clustering resolutions of 1.5 and 0.4 were used, respectively, for dimensional reduction and Louvain clustering.

#### Projection of T-cells from cutaneous sarcoidosis to integrated BAL T-cell embedding

T-cells of a publicly available dataset including three patients suffering from cutaneous sarcoidosis were extracted after preprocessing using the common CellRanger version (v3.1.0) and reference (GRCh38, Ensembl93) as well as quality filtering [31] (Supplementary Table 1). Seurat v4.1.1 was used for mapping of obtained T-cell transcriptomes following the steps detailed in the author’s tutorial [48]. Mapping anchors were identified using the function FindIntegrationAnchors with k.filter = 100, dims = 1:10, and reference.reduction = “pca” (as recommended for unimodal analysis). MapQuery function was run using the identified mapping anchors with reference.reduction = “pca” and T-cell subsets (from Fig. 3C) as refdata for label transfer. Query cells with a label transfer prediction score below 0.4 were labeled as unassigned.

#### Analysis of EMD sample

Raw sequencing data was handled in the same way as described above. Quality filtering thresholds for the number of detected genes, number of detected unique RNA molecules (UMIs) and fraction of mitochondrial UMI-counts are available (Supplementary Table 1). basic Seurat steps (described above) were based on 1000 highly variable genes and 10 first PCA-dimensions. Seurat function CellSelector was used to select MM cell and non-MM cell clusters based on UMAP-coordinates, as unsupervised clustering could not resolve non-MM cell clusters within reasonable clustering resolutions due to their low cell numbers. The described expression of epithelial-associated genes in MM cells was discovered during exploratory data analysis. For gene expression comparison between the EMD sample and intramedullary (IMD) MM samples additional publicly available datasets including one post-CAR T MM case [34] and 13 newly diagnosed MM cases [35] were acquired, quality filtered (Supplementary Table 1), and plasma/MM cells were extracted. Count data was log-normalized. Differential expression analysis results between EMD and IMD can be found in Supplementary Table 2.

#### Annotation of major cell types and subpopulations

Major cell types were annotated based on expression of canonical marker genes (AMp: *CD68*, *CD163*, *FABP4*, *MARCO*; monocytes: *VCAN*, *FCN1*; mDC: *CD1C*, *CD1E*, *FLT3*, *LGALS2*; cDC1: *FLT3*, *LGALS2*, *CLEC9A*, *XCR1*; neutrophils: *FCGR3B*, *CXCL8*, *CSF3R*; *G0S2*; Mast cells: *TPSAB1*, *TPSB2*, *MS4A2*, *GATA2*; T-cells: *CD3D*, *CD3E*, *IL32*; CD4 T-cells: *CD4*; CD8 T-cells: *CD8A*, *CD8B*; Tregs: *FOXP3*, *IL2RA*; NK-cells: *TRDC*, *NKG7*, *KLRC1*, *XCL1*, *XCL2*, *KLRF1;* B-cells: *MS4A1*, *SPIB*; Plasma-cells: *JCHAIN*, *SDC1*, *MZB1*, *TNFRSF17, XBP1*; Fibroblasts: *COL3A1*, *COL2A1*, *MFAP5;* proliferating cells: *MKI67*, *TOP2A*). T-cell subpopulations were annotated upon careful assessment of expressed genes. particularly, expression of canonical lineage and cell state markers was interrogated (Th1: *IFNG*, *TNF*, *TBX21*, *CXCR3*; Th17: *IL17A*, *CCL20*, *RORC*, *CCR6*, *KLRB1*; central memory T-cell (TCM): *CCR7*, *TCF7*, *S1PR1, IL7R, SELL*; naive T-cells: *CCR7*, *TCF7*, *LEF1*; MAIT: *SLC4A10*, *ME1*; cytotoxic lymphocytes (CTL): Granzymes, *PRF1*; CD4 CTL: *CD4*, *CCL4*, *CCL5*, *PRF1*; IFN-responsive: *ISG15*, *MX1, RSAD2*). Macrophage subpopulations were annotated in an analogous fashion (CD163/LGMN Mp: *SPP1*, *LGMN*; pro-inflammatory AMp: *CCL3*, *CCL4*, *CCL20*, *TNFAIP6*, *SOD2*; metallothionein AMp: metallothioneins, e.g. *MT1G*, *MT2A*, *MT1X*; IFN-responsive: *ISG15*, *MX1*, *RSAD2*; Foam cells: *LDLR*, *SQLE*, *HMGCR*, *MSMO1*).

#### UniFrac distance analysis

To test if T-cells from different conditions in the integrated BAL analysis tended to form the same unsupervised clusters, UniFrac distance analysis was applied [30]. Mean principal component (PC) values were calculated across significant PCs for each unsupervised cluster (see details above). Euclidean distance was calculated for each PC between clusters and a distance matrix was built with decreasing weights for PCs, analogous to decreasing eigenvalues of PCs. The distance matrix was used as input for hierarchical clustering with the complete linkage method (R function hclust) to obtain a precalculated tree. Code from the scUniFrac R package was modified to enable use of this precalculated tree for computation of generalized UniFrac distances between the conditions [53, 54] (code available on GitHub).

#### Signature module score calculation

Single-cell level scores for signatures were computed using the Seurat function AddModuleScore. In brief, genes were binned (nbin = 24) based on their average expression in all cells. Expression of genes from a given signature was calculated and subtracted by the aggregated expression of a control feature set (ctrl = 100) selected randomly from the bins the analysed signature genes were part of. Statistical significance was assessed by pairwise, one-sided (alternative = “greater”) Wilcoxon rank-sum test (R function wilcox.test) of each group compared to the average. Signatures were retrieved from the literature. Signatures and respective sources are listed in Supplementary table 1.

#### Differential expression analysis

Differential expression analysis was performed using the Wilcoxon rank-sum test implementation of the Seurat function FindAllMarkers (only.pos = TRUE). Differential expression analysis results are available in Supplementary Table 2.

### Flow cytometry

EMD cells were thawed in RPMI supplemented with 10% FCS. Staining was performed in PBS at 4 °C with the following antibodies: CD184/CXCR4 – PerCP/eFluor710 (1:100, 12G5, Thermofisher), CD326/EpCAM – APC (1:100, 9C4, Biolegend), CD269/BCMA – PE (1:100, REA361, Miltenyi), CD38- FITC (1:100, HIT2, Thermofisher), CD138 – BV510 (1:20, MI15, Biolegend), CD45 – BV605 (1:20, 2D1, Biolegend), CD3 – AF700 (1:100, HIT3a, Biolegend). For dead cell exclusion, Fixable Viability Dye eFluor ™ 780 (1:1000, Thermofisher) was used. A FcGr blocking step was not performed. Events were acquired on the Attune NxT (Thermofisher). Data was analyzed with FlowJo version 10.8.0.

### Statistical analysis

The non-parametric two-sided Wilcoxon rank-sum test was used to compare gene expression values between groups of cells. A *p*-value <0.05 was considered as statistically significant.

## Supplementary information

Supplementary Information

Supplementary Table 1

Supplementary Table 2

## Data Availability

Data generated in the present study are deposited at European Genome-Phenome Archive (EGA) under study ID EGAS00001006133. Healthy BAL datasets from Liao M. et al. [25] are accessible from the Gene Expression Omnibus (GEO) database under accession no. GSE145926. COVID-19 ARDS BAL data from Wendisch et al. [26] are available at the European Genome-Phenome Archive (EGA) under study ID EGAS00001004928. Sarcoidosis BAL data from Liao S.Y. et al. [27] is accessible on GEO under accession no. GSE184735. Data from Damsky et al. [31], including three patients with cutaneous sarcoidosis, is available on GEO with accession no. GSE169147. The dataset of a post-CAR T relapsed multiple myeloma patient from Da Vià et al. [34] can be accessed on GEO with accession no. GSE143317. Newly diagnosed MM plasma cell datasets from de Jong et al. [35] are available on ArrayExpress, no. E-MTAB-9139.

## Code availability

All R code used for scRNA-seq data analysis has been deposited on GitHub: https://github.com/alexleipold/SarcoidosisAfterCART
