# Supplementary material for: Th17.1 cell driven sarcoidosis-like inflammation after anti-BCMA CAR T cells in multiple myeloma
Source: Leukemia. 2023 Jan 31;37(3):650–8. doi: 10.1038/s41375-023-01824-0 (PMC9888347; doi:10.1038/s41375-023-01824-0)
Supplement: Supplementary file 1 — Supplementary Information [file 41375_2023_1824_MOESM1_ESM.docx]

**Th17.1 cell driven sarcoidosis-like inflammation after anti-BCMA CAR T cells in multiple myeloma**

Alexander M. Leipold^1,2^, Rudolf A. Werner^3^, Johannes Düll^4^, Pius Jung^5^, Mara John^2^, Emilia Stanojkovska^2^, Xiang Zhou^4^, Hannah Hornburger^4^, Anna Ruckdeschel^4^, Oliver Dietrich^1^, Fabian Imdahl^1^, Tobias Krammer^1^, Stefan Knop^4^, Andreas Rosenwald^6^, Andreas Buck^3^, Leif Erik Sander^7,8^, Hermann Einsele^4^, K. Martin Kortüm^4^, Antoine-Emmanuel Saliba^1,*^, Leo Rasche^2,4,*^

**Affiliations**

^1^ Helmholtz Institute for RNA-based Infection Research (HIRI), Helmholtz-Center for Infection Research (HZI), Würzburg, Germany

^2^ Mildred Scheel Early Career Center, University Hospital of Würzburg, Würzburg, Germany

^3^ Department of Nuclear Medicine, University Hospital Würzburg, Würzburg, Germany

^4^ Department of Internal Medicine 2, University Hospital of Würzburg, Würzburg, Germany

^5^ Department of Internal Medicine 1, University Hospital of Würzburg, Würzburg, Germany

^6^ Institute of Pathology, University of Würzburg, Würzburg, Germany

^7^  Charité - Universitätsmedizin Berlin, Corporate Member of Freie Universität Berlin and Humboldt-Universität zu Berlin, Department of Infectious Diseases, Respiratory Medicine and Critical Care

^8^ Berlin Institute of Health, Center for Regenerative Therapies

**Supplementary Figures**

Supplementary Figure 1: Whole body PET imaging at baseline and during follow-up using multiple PET tracers along with maximum intensity projection (MIP) on the right and transaxial PET, PET/CT and CT on the left.

Supplementary Figure 2: scRNA-seq analysis of BAL from a MM patient at 3-months follow-up of a CAR T therapy (CART-BAL) in technical duplicates.

Supplementary Figure 3: scRNA-seq analysis of the T-cell compartment of the CART-BAL from a MM patient at 3-months follow up after anti-BCMA CAR T-cell therapy in technical duplicate.

Supplementary Figure 4: Dataset integration.

Supplementary Figure 5: Analysis of integrated scRNA-seq BAL datasets from CART-BAL, Sarcoidosis, healthy control, COVID-19.

Supplementary Figure 6: scRNA-seq analysis of T-cells isolated from the integrated BAL datasets.

Supplementary Figure 7: Projection of single-T-cell transcriptomes from skin sarcoidosis biopsies (Damsky *et al*, 2022) onto the integrated BAL T-cell embedding.

Supplementary Figure 8: scRNA-seq analysis of monocytes/macrophages isolated from the integrated BAL datasets.

Supplementary Figure 9: FACS analysis of EMD sample and comparison of EMD sample scRNA-seq expression of selected genes to publicly available IMD scRNA-seq samples.

**Supplementary Tables Legends**

**Supplementary Table 1: Data quality; Public datasets; QC filtering; Gene signatures. Page 1**, scRNA-seq data quality metrics from CellRanger output of data generated in the present study. ‘Identifier’ shows the name of the library in the manuscript. ‘Library’ shows the formal library name (also used in EGA). **Page 2**, Description of publicly available datasets used for data integration and projection. ‘Reference’ shows an identifier based on the first author of the respective study. ‘Title’ shows the manuscript title. ‘Journal’ indicates where the article was published. ‘Condition’ indicates the underlying disease context. ‘PMID’ and ‘DOI’ show identifiers to access the manuscripts. The database and respective accession numbers (database: accession no.) for the datasets can be found at ‘Access’. **Page 3**, Quality metrics and customized parameters used for quality filtering of all datasets that were used in this study. Parameters were customized in order to account for varying underlying data qualities between studies. ‘Identifier’ indicates the name of the datasets generated in the present study or the first author of the respective study public data is derived from (analogue to ‘Reference’ in Table page 2). ‘Condition’ indicates the underlying disease context. **Page 4**, Description of Th1-polarized Th17 signatures used for module score calculation. ‘Reference’ shows an identifier based on the first author of the respective study. ‘Title’ shows the manuscript title. ‘Cluster’ indicates the subset from the original publication. ‘PMID’ and ‘DOI’ show identifiers to access the manuscripts. ‘Genes’ shows the gene set of the respective signature.

**Supplementary Table 2: Differential expression analysis results. Page 1-11**, Statistics related to results of conducted differential expression analyses. The tables are ordered by cell type (column 1 ‘cluster’). Genes (column 2 for gene symbol & column 8 for ENSEMBL ID) are ordered by increasing p-values (column 6) and decreasing average log-fold change (column 2). Bonferroni-method was used to adjust p-values for multiple comparisons (column 7). Percentages of non-zero expressing cells in the respective ‘cluster’ and all other cells are indicated (column 4 for ‘cluster’ & column 5 for all other cells).


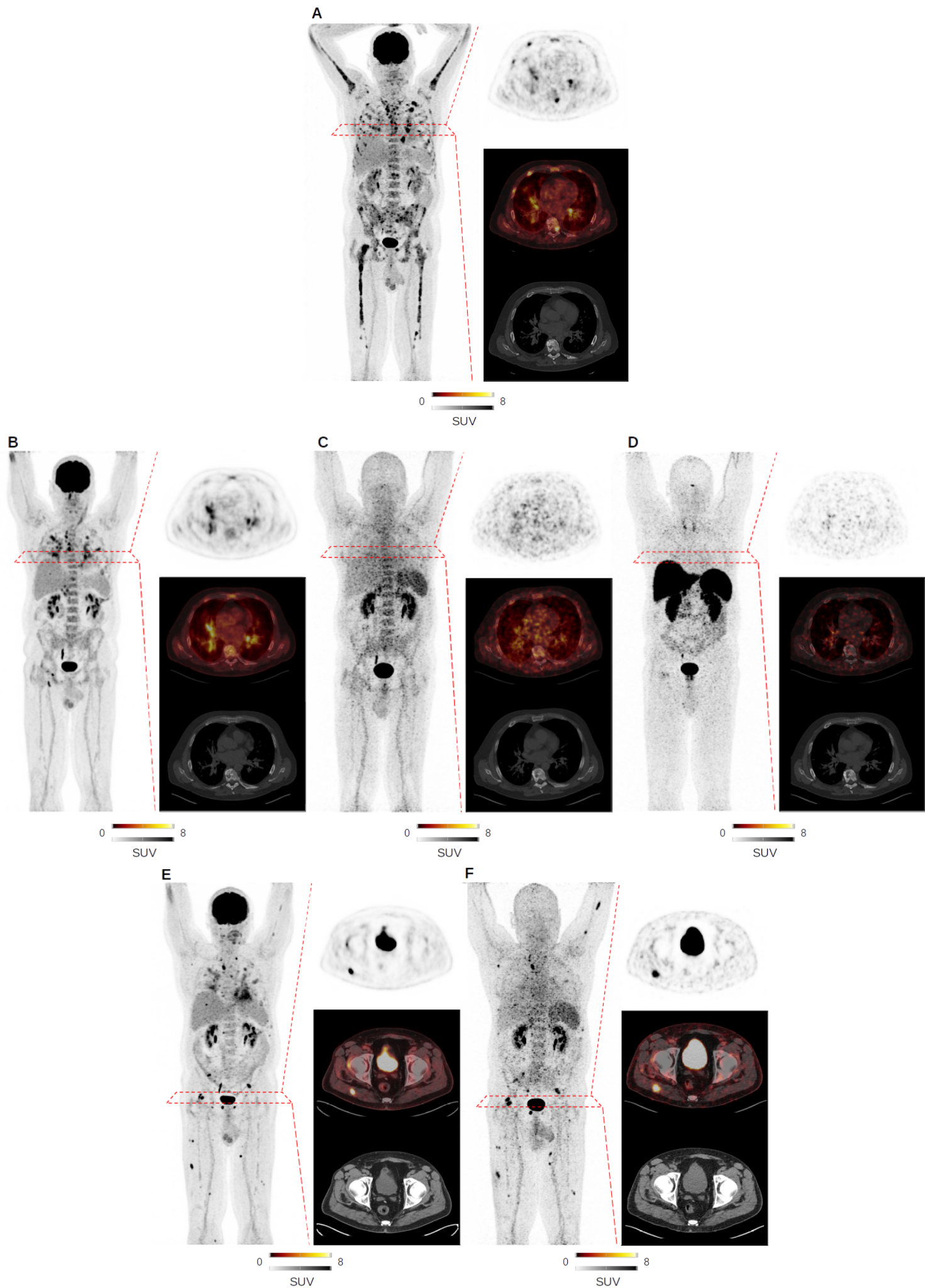


**Supplementary Figure 1: Whole body PET imaging at baseline and during follow-up using multiple PET tracers along with maximum intensity projection (MIP) on the right and transaxial PET, PET/CT and CT on the left. A**, FDG-PET at baseline shows multiple focal lesions at the axial and appendicular skeleton. Respective transaxial slides revealed multiple disease manifestations in the ribs. **B-D**, Follow-up assessment 3 months after CAR T infusion shows full resolution of focal lesions, but residual FDG uptake located to the lung and mediastinal lymph nodes, as visualized on the transaxial slides. Matched simultaneous CXCR4-targeted Pentixafor (**C**) and SSTR-directed DOTATOC (**D**) scans did not show any focal uptake. Respective transaxial slides on the identical site as chosen for FDG scan are shown. **E**,**F**, Matched simultaneous FDG (**E**) and CXCR4-directed Pentixafor (**F**) PET 6 months after CAR T infusion depicted new focal lesions in line with relapse, as seen on transaxial slides at the pelvis.


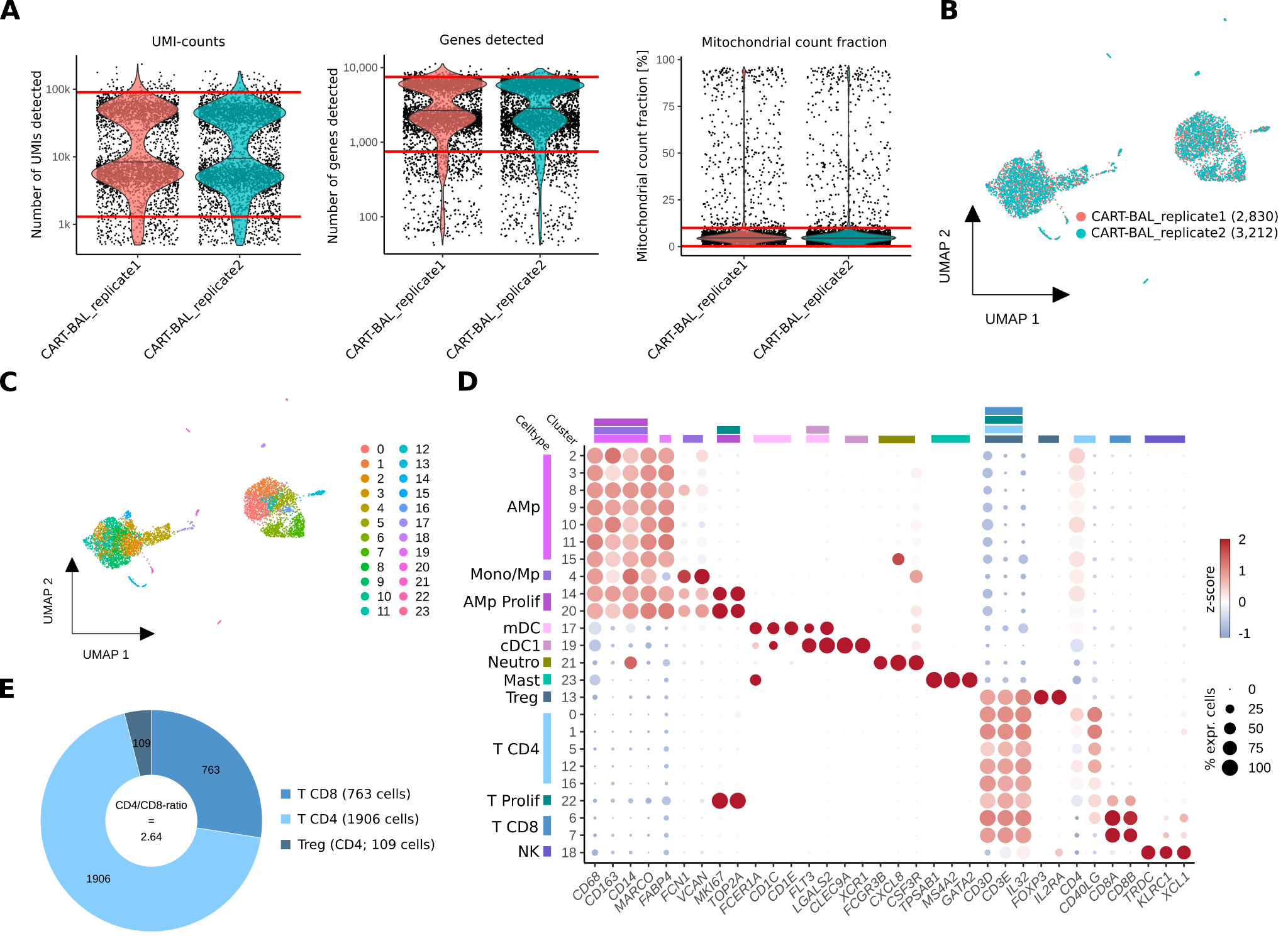

**Supplementary Figure 2: scRNA-seq analysis of BAL from a MM patient at 3-months follow-up of a CAR T therapy (CART-BAL) in technical duplicates. A**, Data quality metrics and filtering across two scRNA-seq CART-BAL technical duplicates depicted in violin plots (replicate1: 3,764 cells, replicate2: 4,180 cells). Red lines indicate the thresholds that were used for quality control filtering. For UMI-counts and genes detected, log10-scale was used. **B**, UMAP embedding visualising scRNA-seq of 6,042 cells from CART-BAL color-coded by technical replicate. Numbers in brackets indicate cell number per replicate after quality control filtering (panel a). **C**, UMAP embedding as in (**B**) partitioned in clusters and color-coded by Louvain cluster. **D**, Dotplot showing log-normalized, scaled expression (color) and the proportion in percentage of non-zero expressing cells (size) of canonical marker genes per cluster identified in the UMAP (panel c). **E**, Pie plot depicting the proportions of CD4 cells, Tregs, and CD8 cells within T-cells, as well as the CD4/CD8-ratio. Abbreviations: AMp - alveolar macrophage; Mono/Mp - monocyte/macrophage; Neutro - neutrophil; Prolif: Proliferating.


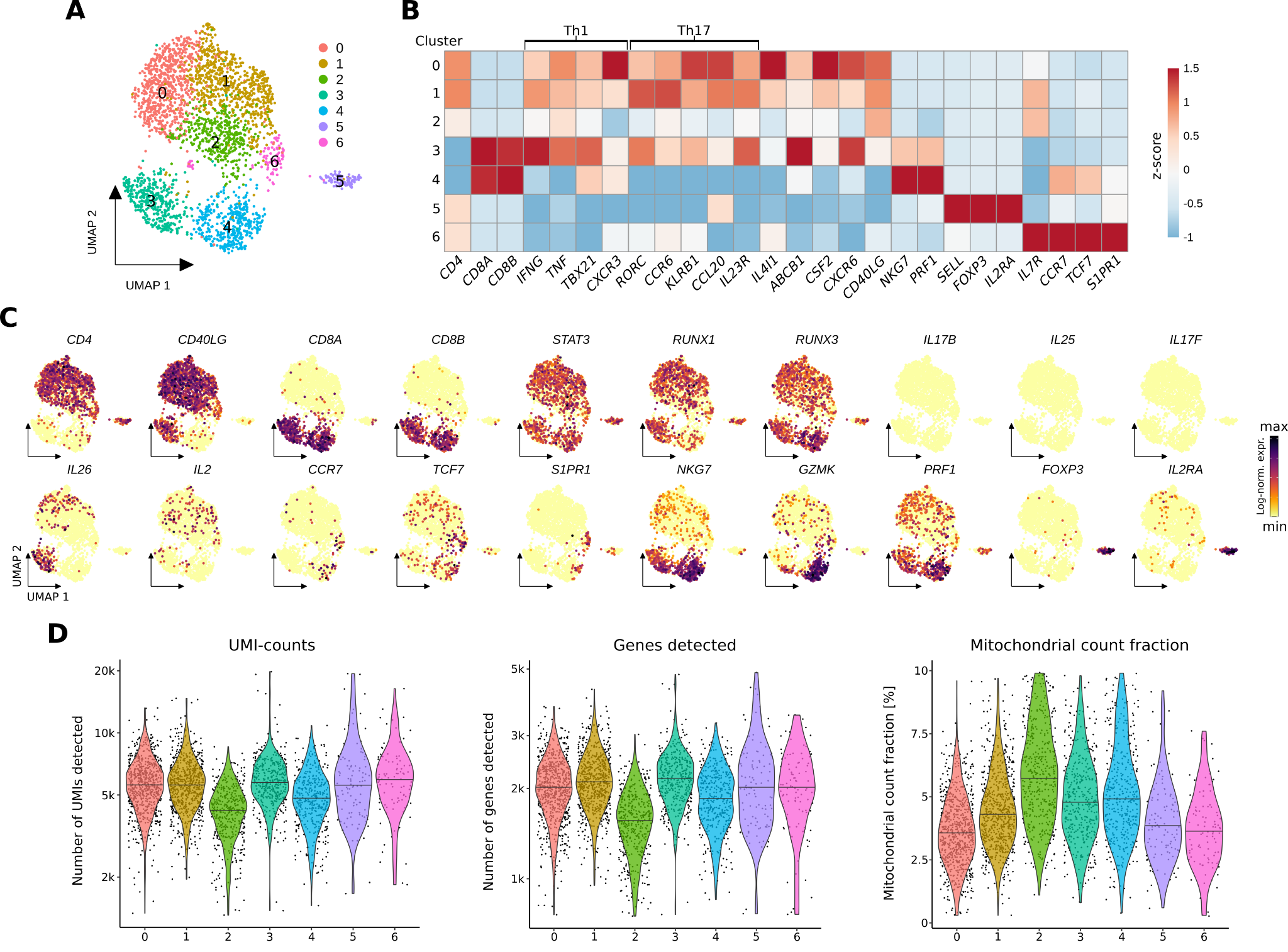

**Supplementary Figure 3: scRNA-seq analysis of the T-cell compartment of the CART-BAL from a MM patient at 3-months follow up after anti-BCMA CAR T-cell therapy in technical duplicate. A**, UMAP embedding of single-cell transcriptomes of T-cells from the CART-BAL colored and numbered by Louvain cluster. **B**, Heatmap showing the z-score of mean log-normalized expression of selected genes per cluster identified in panel a. Th1- and Th17-associated genes are indicated by brackets. **C**, Log-normalized gene expression of selected T-cell markers color-coded and projected on the UMAP embeddings from (**A**). **D**, Violin plots depicting UMI-counts, number of genes detected and percentage of mitochondrial genes across the T-cell clusters identified in (**A**).


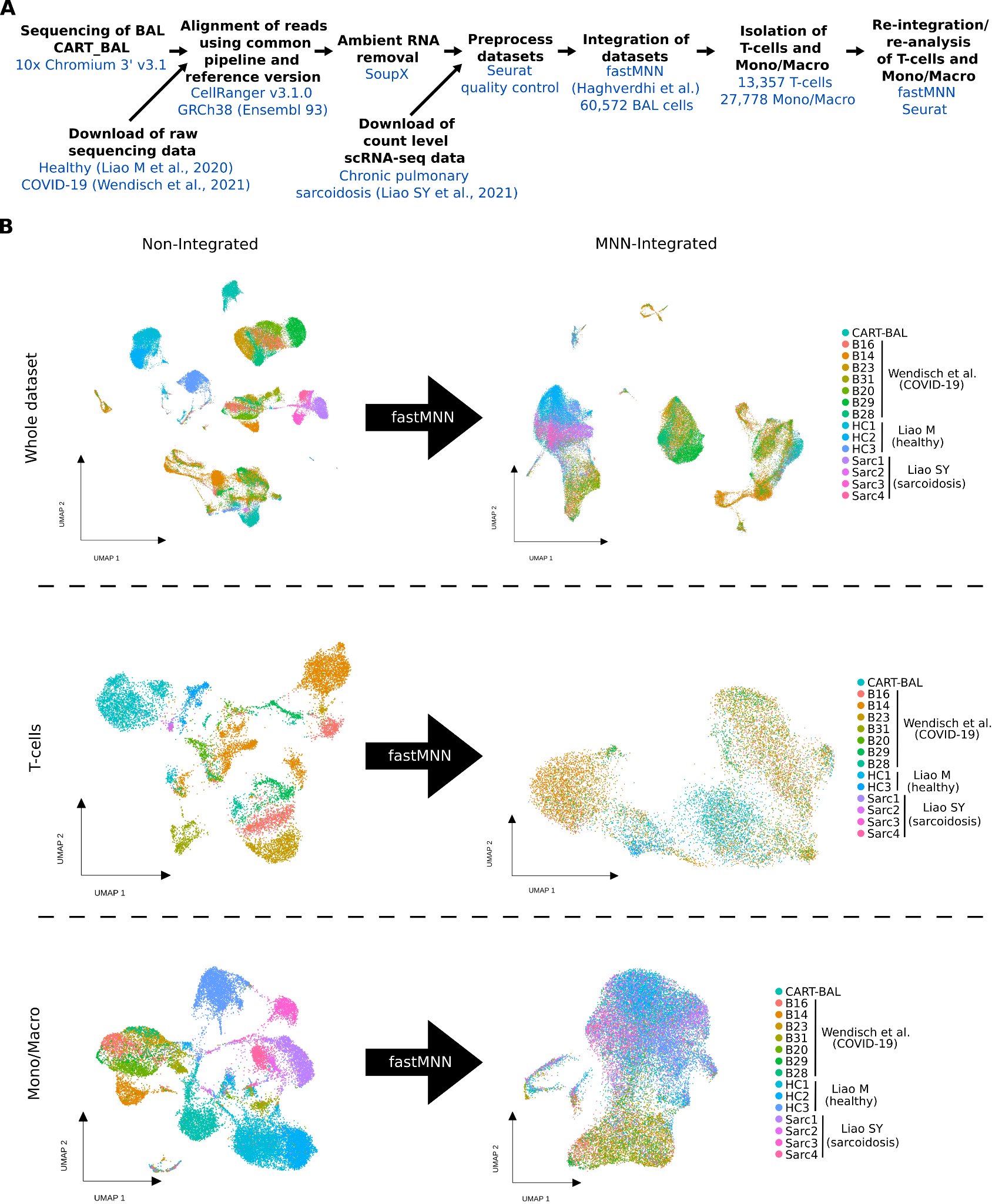


**Supplementary Figure 4: Dataset integration. A**, Schematic of the workflow used for data integration. **B**, UMAP embeddings of the merged BAL datasets with and without MNN-integration colored by patient or donor for the whole dataset (60,572 cells), T-cells (13,357 cells) and monocyte/macrophages (Mono/Macro; 27,778 cells). Abbreviations: BAL - bronchoalveolar lavage.


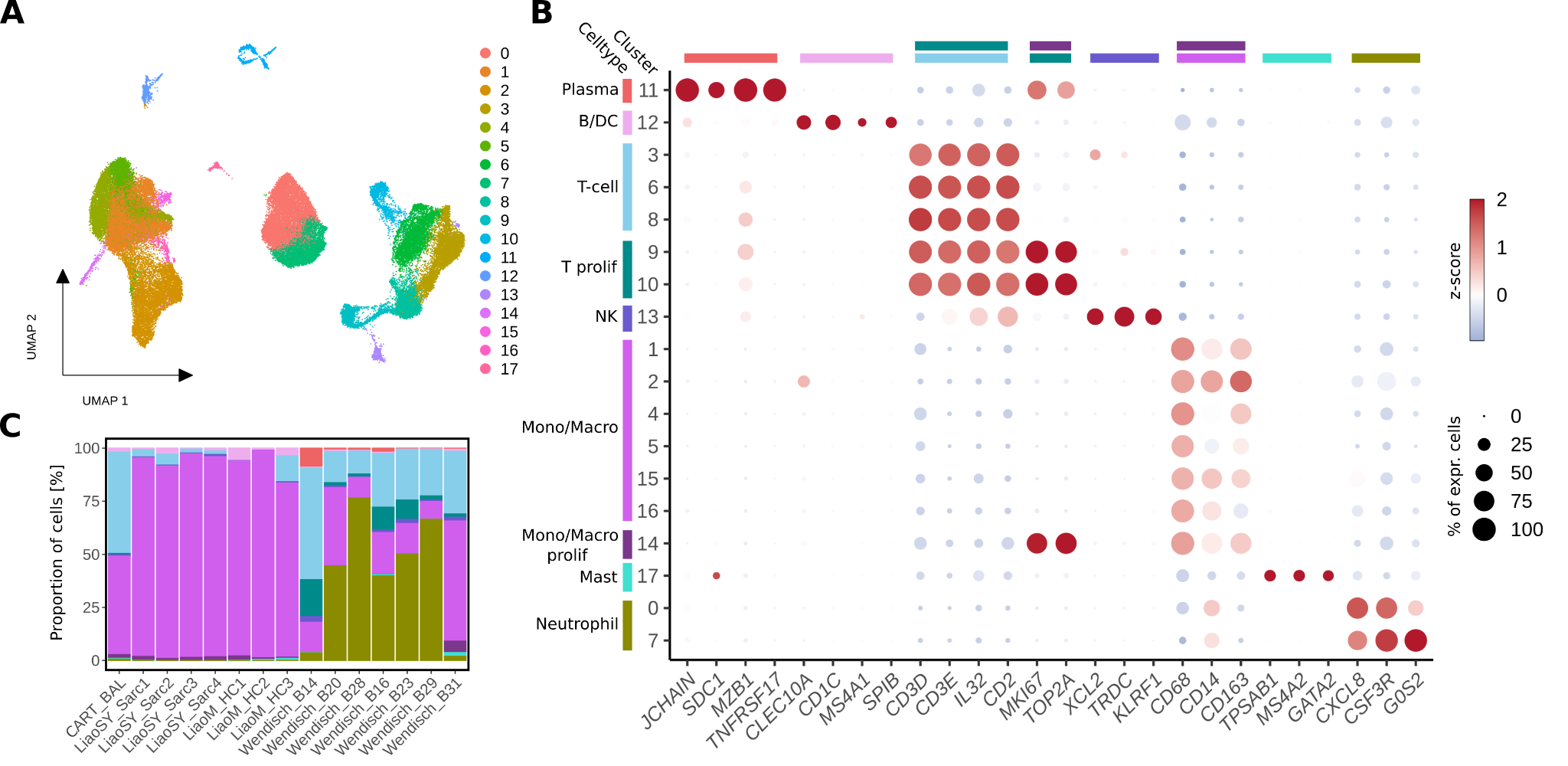

**Supplementary Figure 5: Analysis of integrated scRNA-seq BAL datasets from CART-BAL, Sarcoidosis, healthy control, COVID-19. A**, UMAP embedding of integrated BAL cells obtained after data integration (**Supplementary Fig 4B**) colored by the Louvain cluster. **B**, Dotplot showing log-normalized, scaled expression (color) and the proportion in percentage of non-zero expressing cells (circle size) of canonical marker genes per cluster and annotated cell types. **C**, Relative proportions of cell types across patients and donors (Liao S.Y. et al. - sarcoidosis; Liao M. et al. - healthy control; Wendisch et al. - Covid-19). Cell Types are color coded as in (**B**). Abbreviations: Mono/Macro - monocyte/macrophage; prolif – proliferating.


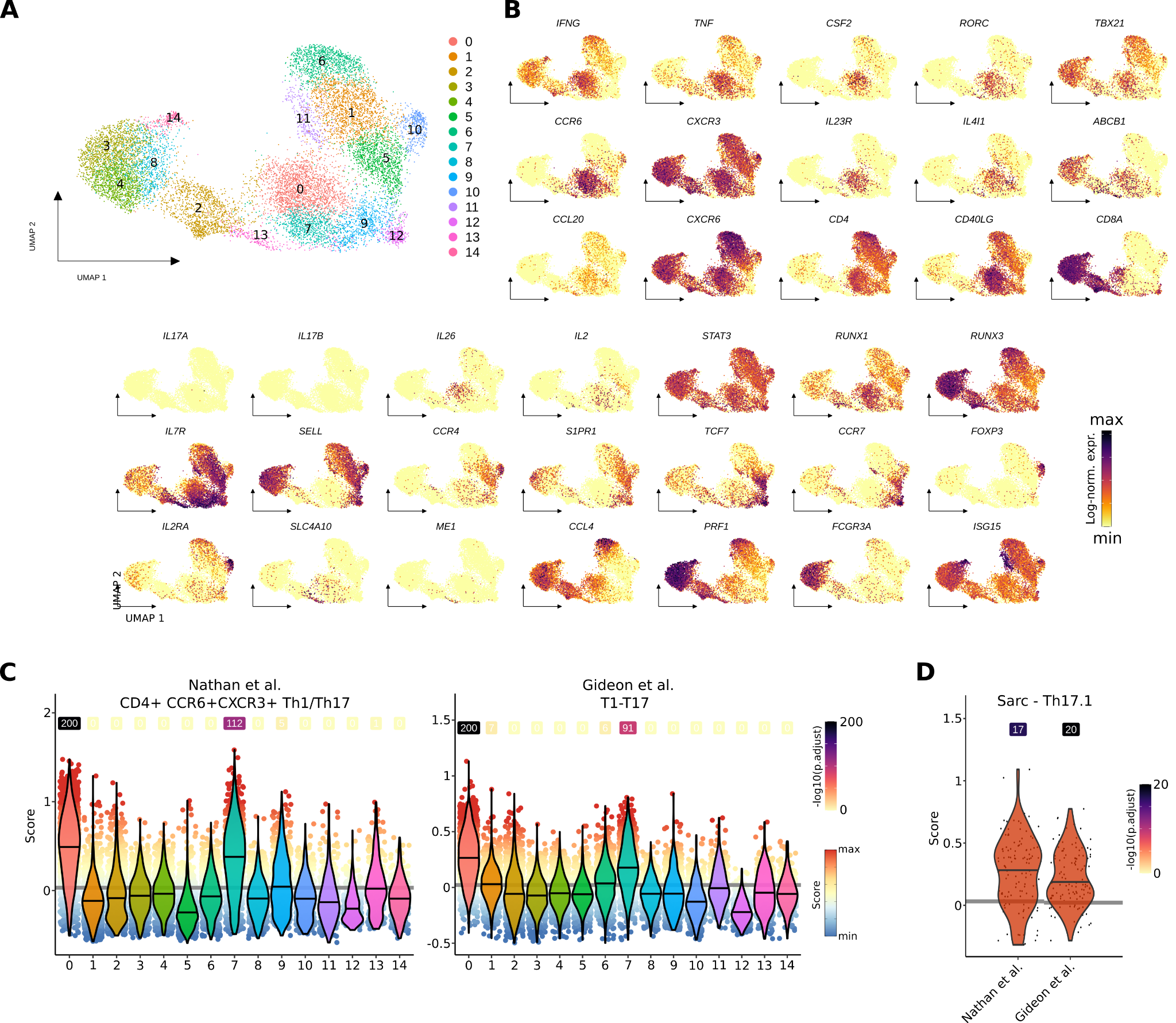

**Supplementary Figure 6: scRNA-seq analysis of T-cells isolated from the integrated BAL datasets. A**, UMAP embedding of T-cells (T-cells, **Fig 3B and Supplementary Fig 4B**) from integrated BAL datasets of four conditions colored by Louvain clusters. **B**, Gene expression level of selected genes log-normalized, color-coded and projected on the UMAP embeddings of T-cells from integrated BAL datasets. **C**, Cell-based gene set module scores of two Th1-polarized Th17 gene signatures depicted as violin plots across Louvain clusters. Lines in violins show median scores per cluster. Grey lines indicate the average scores across all T-cells. Dot color specifies the signature module score and numbers specify -log10 transformed adjusted p-values (one-sided Wilcoxon rank-sum test against the average; -log10(p.adjust) with value ‘infinite’ (p.adjust = 0) were set to 200). **D**, Scores from (**C**) for Th17.1 cells (see **Fig 3C**) from the condition sarcoidosis (Sarc). Lines in violins show median scores per signature. Grey lines indicate the average scores across all T-cells for the respective signature. Numbers specify -log10 transformed adjusted p-values (one-sided Wilcoxon rank-sum test against the average of all T-cells). Abbreviations: TCM - central memory T-cell; CTL - cytotoxic T-lymphocyte.


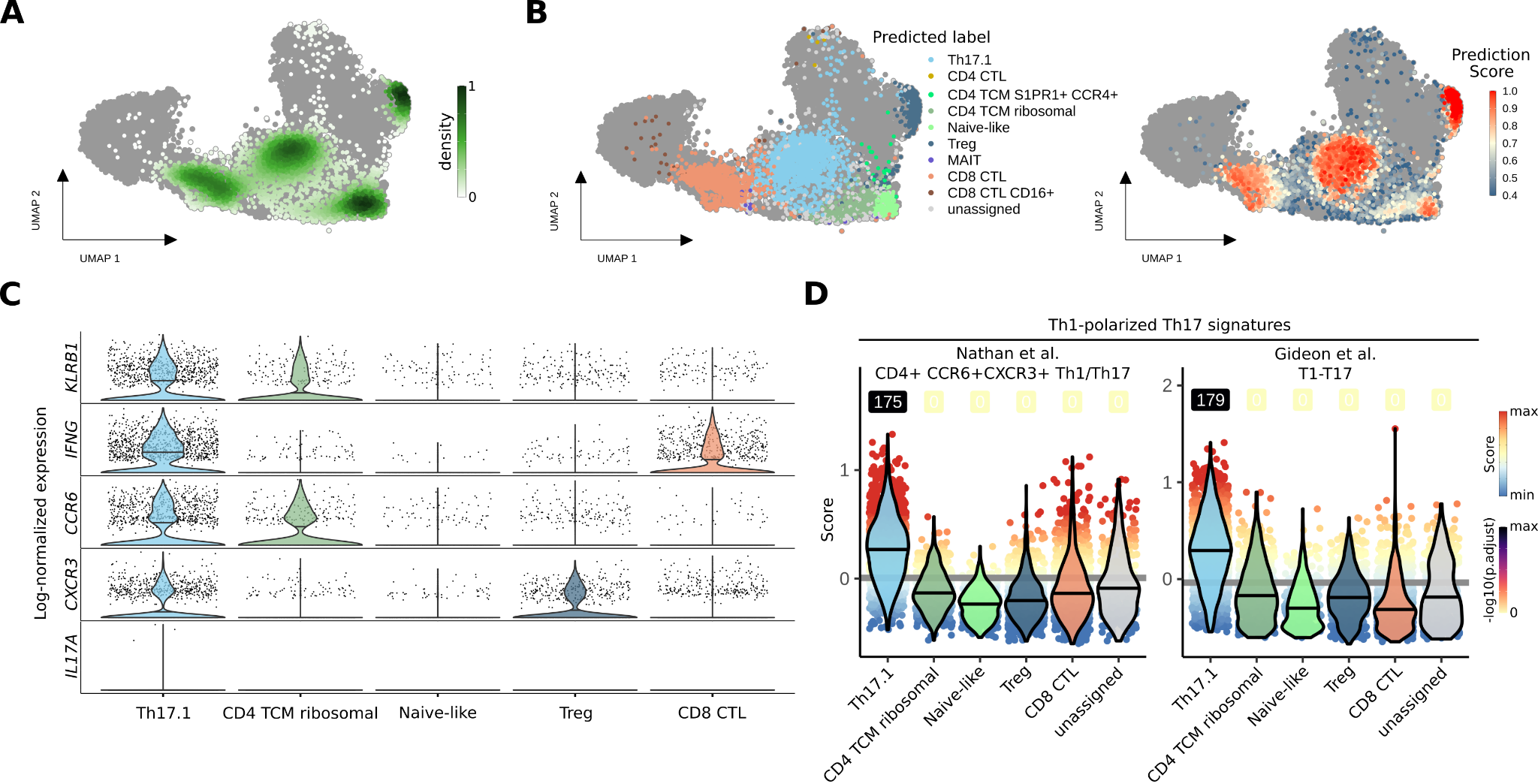

**Supplementary Figure 7: Projection of single-T-cell transcriptomes from skin sarcoidosis biopsies (Damsky *et al*, 2022) onto the integrated BAL T-cell embedding. A**, Overlay of kernel density estimation of the skin sarcoidosis T-cells on the integrated BAL T-cell UMAP embedding. **B**, Label transfer predictions (left) and prediction scores (right) as overlay on the integrated BAL T-cell UMAP embedding. **C**, Depiction of log-normalized expression of selected genes in skin sarcoidosis T-cells as violin plots across predicted T-cell subsets. Predicted T-cell subsets with more than 30 assigned cells are shown. **D**, Cell-based gene set module scores of two Th1-polarized Th17 gene signatures in skin sarcoidosis T-cells depicted as violin plots across predicted T-cell subsets. Lines in violins show median scores per predicted T-cell subset. Grey lines indicate the average scores across all projected T-cells. Dot color specifies the signature module score and numbers specify -log10 transformed adjusted p-values (one-sided Wilcoxon rank-sum test against the average). Predicted T-cell subsets with more than 30 assigned cells and unassigned cells are shown. Abbreviations: CTL - cytotoxic T-lymphocyte; TCM - central memory T-cell; MAIT - mucosal associated invariant T-cell.


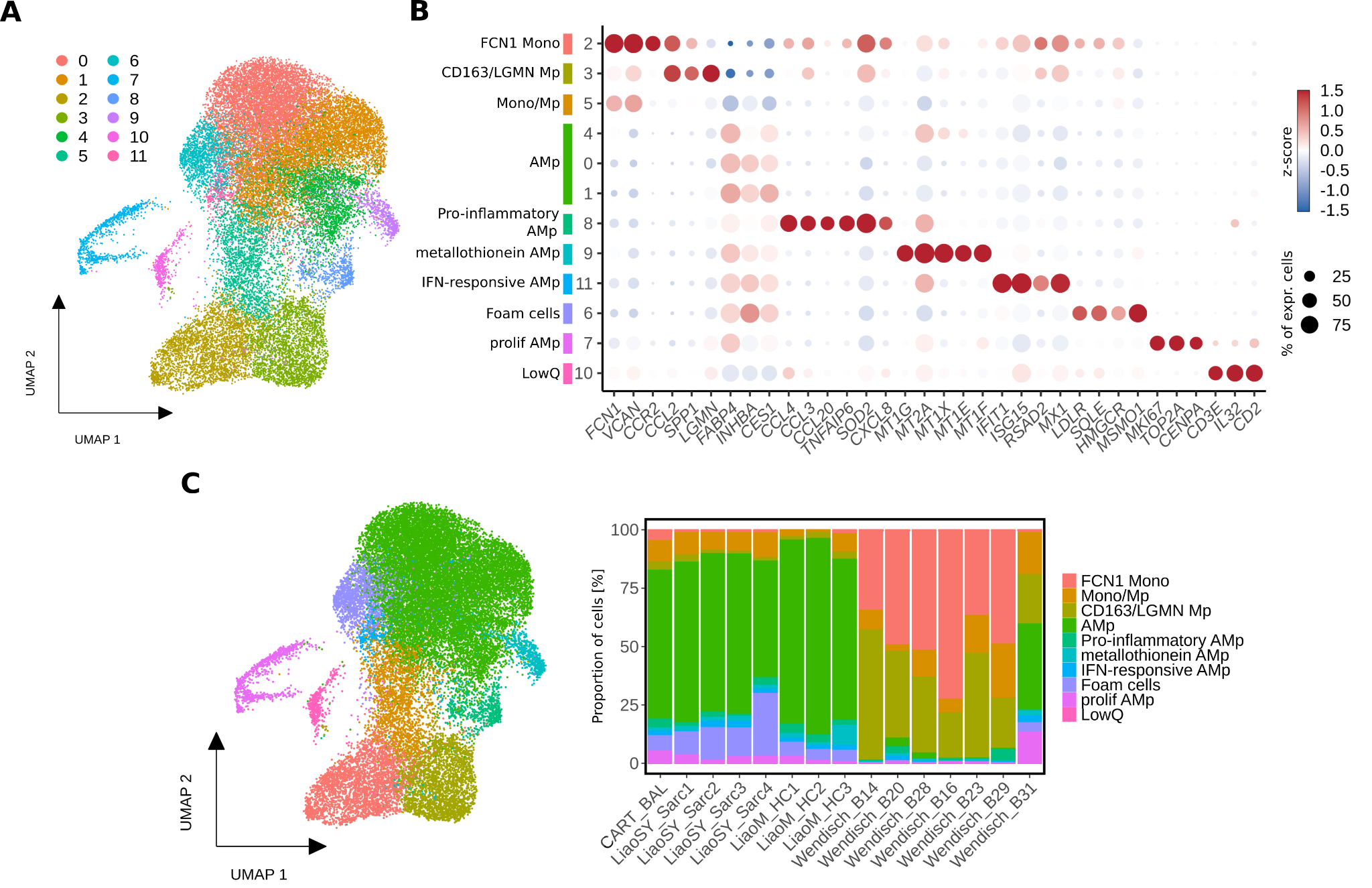

**Supplementary Figure 8: scRNA-seq analysis of monocytes/macrophages isolated from the integrated BAL datasets. A**, UMAP embedding of 27,778 monocytes/macrophages (Mono/Macro, **Fig 3B and Supplementary Fig 4B**) from integrated BAL datasets of four conditions colored by Louvain clusters. **b**, Dotplot showing log-normalized, scaled expression (color) and the proportion in percentage of non-zero expressing cells (size) of canonical marker genes per cluster. Respective subset annotation with color code is shown as colored bars (left). **c**, Mono/Macro subset annotation plotted as UMAP embedding and proportion bar plot over individual patients/donors. Abbreviations: Mono - monocyte; Mp - macrophage; AMp - alveolar macrophage; LowQ - low quality cell.


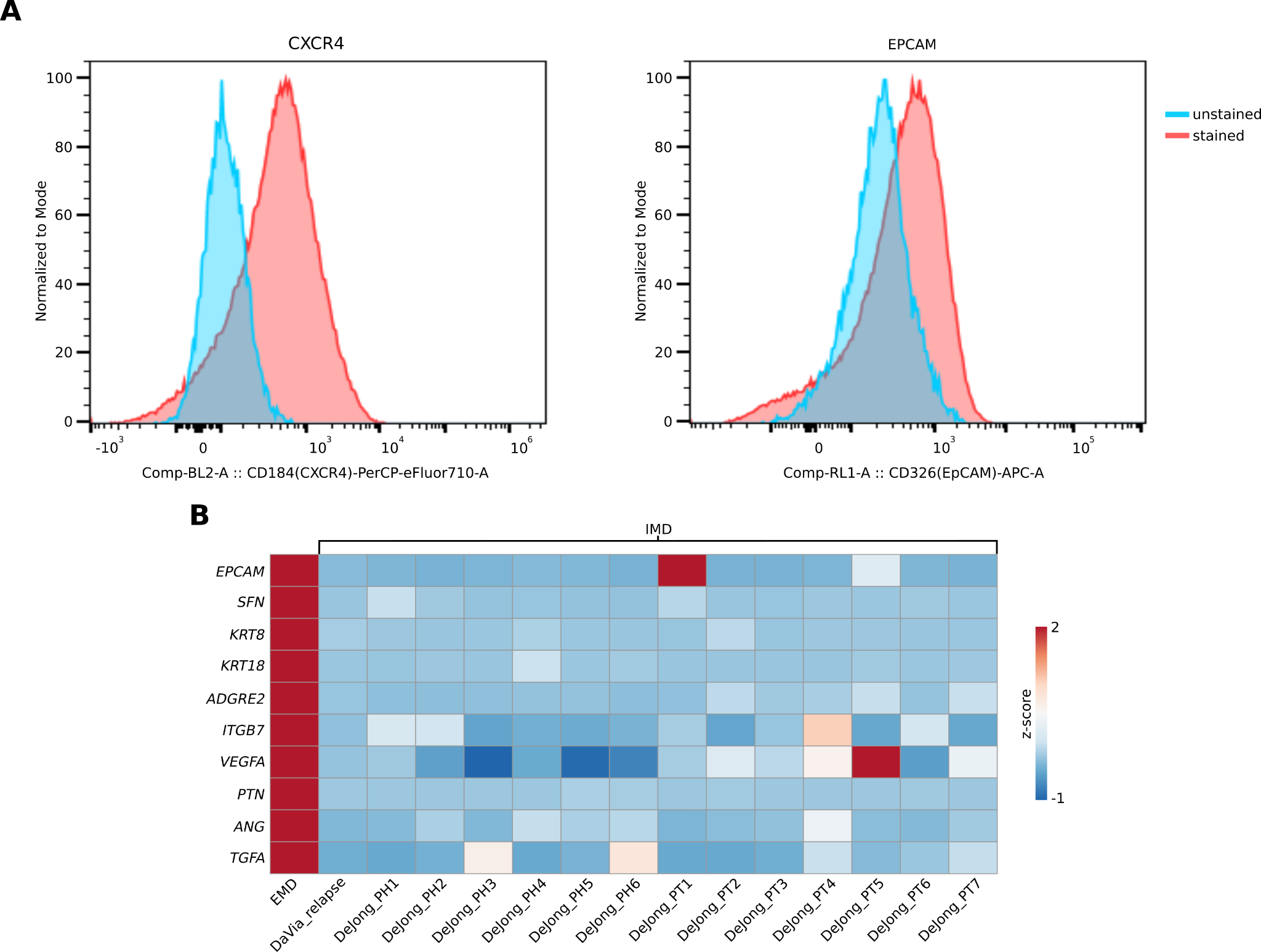

**Supplementary Figure 9: FACS analysis of EMD sample and comparison of EMD sample scRNA-seq expression of selected genes to publicly available IMD scRNA-seq samples. A**, CXCR4 and EPCAM surface protein expression in cells of an extramedullary lesion of our patient at 6 month follow-up. **B**, Heatmap showing the comparison of expression of selected genes between scRNA-seq data of the extramedullary lesion of our patient at 6 month follow-up and publicly available IMD scRNA-seq datasets. Selected genes are epithelial- (*EPCAM*, *SFN*, *KRT8*, *KRT18*), extracellular matrix interaction- (*ADGRE2*), cell-cell adehsion- (*ITGB7*), as well as angiogenesis-associated (*VEGFA*, *PTN*, *ANG*) genes and *TGFA*. Differential expression analysis results between EMD and IMD can be found in **Supplementary Table 2**. Abbreviations: EMD - extramedullary MM disease; IMD - intramedullary MM disease.
